# Temperature management of patients with sepsis and inflammation in Australian and New Zealand ICUs: a point prevalence study

**DOI:** 10.1186/cc10386

**Published:** 2011-10-27

**Authors:** NE Hammond, MK Saxena, C Taylor, I Seppelt, P Glass, J Myburgh

**Affiliations:** 1 The George Institute for Global Health, Sydney, NSW, Australia; 2 St George Clinical School, University of NSW, Sydney, NSW, Australia; 3 St George Hospital, Sydney, NSW, Australia; 4 Sydney Medical School, University of Sydney, NSW, Australia

## Introduction

The use of pharmacological and physical antipyretic therapies to reduce fever in febrile patients is common in hospital settings. Actual evidence on the frequency of antipyretic use is limited, however, both in general hospital populations and, more specifically, in adult intensive care [1-3]. We undertook a prospective point prevalence study with the aim of identifying the prevalence of physical and pharmacological antipyretic therapies in intensive care patients with sepsis and inflammation. We also recorded the indication for antipyretic therapies, temperature measurement site, and mean temperatures on the study day.

## Methods

We conducted a single-day observational point prevalence study in 38 ICUs in Australia and New Zealand. All patients in participating ICUs at a 10:00 am census point were studied. Data were collected for the 24-hour study day that included the 10:00 am time point.

## Results

**Figure 1:**
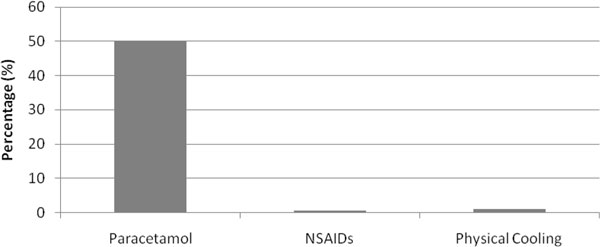
**Type of antipyretic and physical cooling used on the study day (*n *= 311)**.

**Figure 2:**
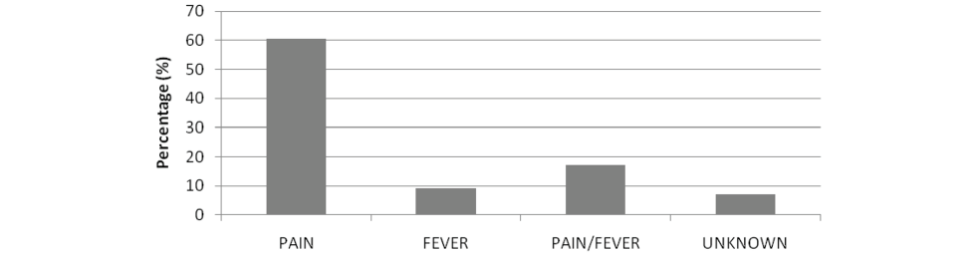
**Indication for paracetamol administration (*n *= 152)**.

**Figure 3:**
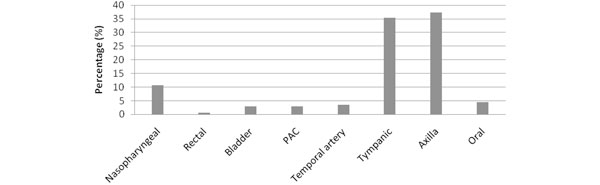
**Temperature measurement site for patients with sepsis and inflammation (*n *= 311)**. PAC, pulmonary artery catheter.

We studied 506 patients, with a mean age 59 years (SD = 17 years); 65% male; APACHE II score 17 (SD = 7), 28-day mortality 14%. Eighty percent of the ICU admissions were unplanned. Of the 506 patients, 311 patients had sepsis and inflammation with mean peak temperature of 37.3°C (SD = 0.8°C). Of these, 35% (*n *= 100/311) had a mean peak temperature above 38°C. In the 24-hour period, paracetamol was used 50% (*n *= 152/311) of the time, nonsteroidal anti-inflammatory drugs (NSAIDs) 0.6% (*n *= 2/311) and physical cooling 1% (*n *= 3/311) (Figure 1). Of patients that had an indication for paracetamol recorded, 64% was for pain (*n *= 92/152), 18% for both pain and fever (*n *= 26/152); and 10% for fever alone (*n *= 14/152) (Figure 2). Sixty-four percent (*n *= 92/152) of the patients who had paracetamol were prescribed regular paracetamol and 36% (*n *= 51/143) had a PRN order. Of the 40 patients who received paracetamol for an indication of fever, the mean peak temperature was 38.3°C (SD = 0.8°C; range 36.1 to 40.2°C). Of the three patients who received physical cooling, the mean peak temperature was 39.2°C (SD = 0.9°C; range 38.5 to 40.2°C). Temperature measurement sites were mainly noncore (*n *= 251/311) with axillary (37%; *n *= 116/311) and tympanic (35%; *n *= 110/311) most common (Figure 3).

## Conclusion

This point prevalence study of intensive care patients with sepsis and inflammation identified pharmacological antipyretics are used regularly for pain management rather than fever management, with paracetamol the most common therapy. The use of physical cooling was rare, and noncore temperature measurements were common. These results are important in understanding current temperature management practice in intensive care and will aid in designing future clinical trials on the subject.
